# HDAC inhibitors overcome immunotherapy resistance in B-cell lymphoma

**DOI:** 10.1007/s13238-020-00694-x

**Published:** 2020-03-11

**Authors:** Xiaoguang Wang, Brittany C. Waschke, Rachel A Woolaver, Samantha M. Y. Chen, Zhangguo Chen, Jing H. Wang

**Affiliations:** Department of Immunology and Microbiology, University of Colorado Anschutz Medical Campus, School of Medicine, 12800 E. 19th Ave, Mail Stop 8333, Aurora, CO 80045 USA

**Keywords:** cancer immunotherapy, HDAC inhibitor, B-cell lymphomas, anti-PD1 resistance, tumor immunogenicity

## Abstract

Immunotherapy has been applied successfully to treat B-cell lymphomas in preclinical models or clinical settings. However, immunotherapy resistance is a major challenge for B-cell lymphoma treatment. To overcome this issue, combinatorial therapeutic strategies have been pursued to achieve a better efficacy for treating B-cell lymphomas. One of such strategies is to combine immunotherapy with histone deacetylase (HDAC) inhibitors. HDAC inhibitors can potentially increase tumor immunogenicity, promote anti-tumor immune responses, or reverse immunosuppressive tumor environments. Thus, the combination of HDAC inhibitors and immunotherapy has drawn much attention in current cancer treatment. However, not all HDAC inhibitors are created equal and their net effects are highly dependent on the specific inhibitors used and the HDACs they target. Hence, we suggest that optimal treatment efficacy requires personalized design and rational combination based on prognostic biomarkers and unique profiles of HDAC inhibitors. Here, we discuss the possible mechanisms by which B-cell lymphomas acquire immunotherapy resistance and the effects of HDAC inhibitors on tumor cells and immune cells that could help overcome immunotherapy resistance.

## Introduction

Non-Hodgkin lymphoma (NHL) is one of the most common type of cancers worldwide, accounting for ~3% of all cancer types (Bray et al., 2018). Among NHL, B-cell lymphomas account for >90% of cases (Scott and Gascoyne, 2014). Current treatments for B-cell lymphomas include surgery, chemotherapy, radiotherapy and immunotherapy. Taking advantage of the immune system to fight cancer, immunotherapy is a relatively newer type of cancer treatment that has the potential to be highly precise and personalized as well as more effective than other types of cancer therapies. Nowadays, immunotherapy is widely used for treating B-cell lymphomas, including antigen specific monoclonal antibodies (e.g., anti-CD20), immune checkpoint inhibitors (ICIs) and chimeric antigen receptor (CAR) T cells.

**Figure 1:**
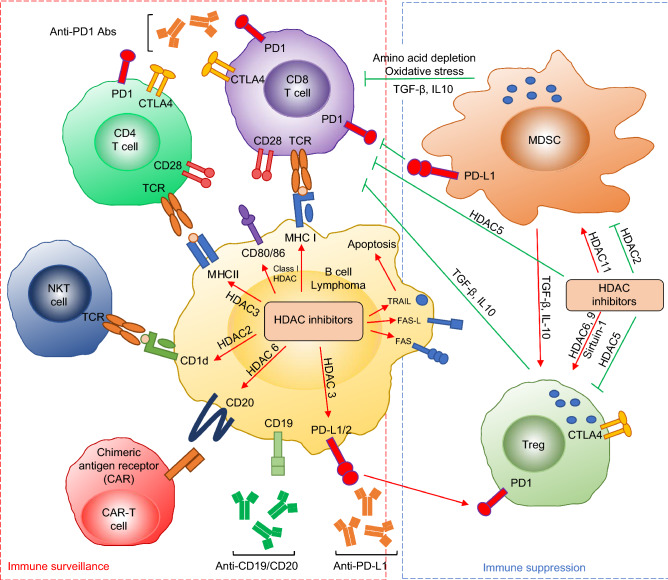
The interaction between HDAC inhibitors and immune system in B-cell lymphoma. Red arrow means increase or activation and green arrow means inhibition

The majority of B-cell lymphomas originate from germinal center (GC) or post-GC B cells, behaving like mature phenotypes (Perez-Duran et al., 2007). Within GCs, B cells undergo secondary antibody gene diversification including class switch recombination (CSR) and somatic hypermutation (SHM) (Wang, 2013; Chen and Wang, 2014). Both CSR and SHM require an enzyme called activation-induced deaminase (AID) (Muramatsu et al., 2000). B-cell lymphomas often harbor reciprocal chromosomal translocations that juxtapose the immunoglobulin (*Ig*) loci and proto-oncogenes (e.g., *c-myc*) (Wang et al., 2008; Wang et al., 2009), or mutations of non-Ig genes that are targets of AID (Kuppers, 2005; Chen and Wang, 2014). In addition, various Ig-derived neoantigens were identified in multiple subtypes of B-cell lymphomas and such neoantigen presentation by major histocompatibility complex (MHC) class I and class II is a general phenomenon of B-cell malignancies (Khodadoust et al., 2017; Khodadoust et al., 2019). As a cancer type with relatively high mutation burden, B-cell lymphomas seem to be highly immunogenic and suitable for cancer immunotherapy (Yi et al., 2018). Moreover, PD-1 expression of tumor-infiltrating T cells is frequently increased in B-cell lymphomas, which provides a biological basis for the application of ICIs (Xu-Monette et al., 2018). However, a majority of B-cell lymphoma patients do not benefit from ICIs (e.g., anti-PD-1) (primary resistance) and others relapse after initial responses (acquired resistance) (Merryman et al., 2017; Xu-Monette et al., 2018). A better understanding of resistance mechanisms of PD-1 blockade is critical to improve the efficacy of cancer immunotherapy and to identify complementary or alternative approaches to overcome immunotherapy resistance in B-cell lymphomas. Hence, this review focuses on discussing the possible mechanisms by which B-cell lymphomas acquire immunotherapy resistance and the effects of HDAC inhibitors on tumor cells and immune cells that could help overcome immunotherapy resistance (Fig. 1).

## Immunotherapy Resistance By B-Cell Lymphomas

While the primary function of B cells is to produce antibodies that mediate humoral immunity, B cells can also function as professional antigen-presenting cells (APCs). B cells constitutively express MHC class I and class II molecules that present antigens to CD8 and CD4 T cells, respectively. Thus, B-cell lymphomas are unique among cancers in that tumor cells can express both MHC class I and class II as APCs (de Charette et al., 2016). To minimize the recognition by T cells and achieve immune evasion during lymphomagenesis, B-cell lymphomas commonly downregulate MHC class I and class II (Xu-Monette et al., 2018). For instance, more than 50% of diffuse large B-cell lymphoma (DLBCL) and 60% of classical Hodgkin lymphoma (HL) present the loss of MHC class I expression (Challa-Malladi et al., 2011; de Charette and Houot, 2018), and diminished MHC class II expression also frequently occurs not only in DLBCL but also in other types of mature B-cell lymphomas (Rimsza et al., 2004; Roberts et al., 2006; Diepstra et al., 2007a; Fangazio et al., 2014; de Charette et al., 2016; Nijland et al., 2017). We recently established a unique mouse model by lineage-specific deletion of a DNA repair gene, *Xrcc4*, and *Trp53* in GC B cells (Chen et al., 2016). This mouse strain spontaneously develops mature B-cell lymphomas, termed G1XP lymphomas (Chen et al., 2016). G1XP lymphomas resemble the key features of human B-cell lymphomas including reciprocal chromosomal translocations and elevated expression of *c-myc* (Chen et al., 2016) and downregulation of MHC class I and class II expression (Wang et al., 2019). Downregulation or loss of MHC class I reduces tumor immunogenicity, decreases the percentage of CD8 and CD4 tumor infiltrating lymphocytes (TILs) and causes resistance to immunotherapy, which correlates to poor prognosis and patient survival (Garrido et al., 2016). Defects in MHC class II expression are associated with reduced T cell infiltration (Rimsza et al., 2004) and inferior survival in patients of DLBCL, primary mediastinal B-cell lymphoma (PMBCL) or HL (Rimsza et al., 2004; Roberts et al., 2006; Diepstra et al., 2007b), as well as poor prognosis in patients of DLBCL and PMBCL following different chemotherapy regimens (Rosenwald et al., 2002; Rimsza et al., 2004; Roberts et al., 2006; Rimsza et al., 2007; Rimsza et al., 2008).

There are two types of MHC down-regulation: irreversible genetic alterations (‘hard lesions’) and reversible epigenetic changes (‘soft lesions’) (Garrido et al., 2010). Comparing with irreversible alterations, reversible downregulation of MHC is usually mediated by epigenetic modifications (Garrido et al., 2010). In human cancers, reversible downregulation dominates the defects in MHC class I expression (Smahel, 2017). Notably, reversible downregulation of MHC class II is mediated by decreased histone acetylation rather than DNA hyper-methylation in DLBCLs (Cycon et al., 2013). Since antigen presentation by tumor cells in the context of MHCs is generally regarded as a prerequisite for effective cancer immunotherapy (Nijland et al., 2017), downregulation of MHC expression represents a contributing factor in immunotherapy resistance (Sharma et al., 2017). Our recent studies show that B-cell lymphomas with low MHC expression resist PD-1 blockade; furthermore, upregulating MHC expression sensitizes B-cell lymphomas to PD-1 blockade (Wang et al., 2019). While HLs frequently reduce MHC class I expression, HLs exhibit a high response rate to PD-1 blockade (Roemer et al., 2016; Ok and Young, 2017), suggesting that the therapeutic effect of PD-1 blockade may not be only restricted to MHC class I-dependent CD8 T cell-mediated killing. In this regard, HLs generally express more MHC class II than MHC class I and are enriched for contact with CD4 T cells rather than CD8 T cells, which indicates that MHC class II may play a significant role in mediating responses to PD-1 blockade (Carey et al., 2017). Consistently, our data support that increased MHC class II contributes to the therapeutic effects of PD-1 blockade (Wang et al., 2019).

CD20 is expressed on the surface of B cells starting from late pro-B cells through memory B cells, but not on either early pro-B cells or plasma blasts and plasma cells (Murphy and Weaver, 2017). CD20 is also expressed on the surface of neoplastic B cells (Olejniczak et al., 2006). Several chimeric monoclonal anti-CD20 antibodies were developed to target CD20 for treating B-cell lymphomas (Maloney, 2012). However, multiple mechanisms may underlie resistance to anti-CD20 therapy. Firstly, CD20 expression varies considerably between different lymphoma subtypes or within a given subtype, which correlates with clinical responses to anti-CD20 (Olejniczak et al., 2006; Johnson et al., 2009). Secondly, a gradual loss of CD20 surface expression is detected in neoplastic B cells with repeated exposure to anti-CD20 antibody (Hiraga et al., 2009; Tsai et al., 2012). Thirdly, epigenetic mechanisms may also contribute to the downregulation of CD20 expression upon anti-CD20 treatment (Hiraga et al., 2009). Recently, CAR T cell immunotherapy against CD20 or CD19 has been developed to treat relapsed or refractory B-cell malignancies (Zhou et al., 2018). Despite the impressive remission rates of CAR T cell therapy, some patients develop initial resistance or relapse upon this novel therapy (Park et al., 2018). The resistant mechanisms of CAR T cell therapy have been extensively reviewed elsewhere (Cheng et al., 2019). For the CAR T cell-treated relapsed B-cell lymphoma patients, tumors can escape the recognition of CAR T cells by losing the very antigens targeted by CAR T cells (Shalabi et al., 2018; Shah et al., 2019). We summarize the current approved immunotherapeutic agents for treating B-cell lymphomas (Table 1), including monoclonal antibodies, antibody-drug conjugates (e.g., Brentuximab vedotin and polatuzumab vedotin-piiq (Polivy)) (Chau et al., 2019), immune checkpoint inhibitors and CAR T cells (Shah et al., 2019).

**Table 1:** Approved immunotherapeutic agents for B-cell lymphomas

Therapy type	Name	Target	Subtype	Year
Monoclonal antibodies	Rituximab	CD20	NHL	1997
	Obinutuzumab	CD20	FL	2016
	Brentuximab vedotin	CD30	Relapsed or refractory HL	2011
	Polatuzumab vedotin-piiq	CD79b	DLBCL	2019
Immune checkpoint inhibitors	Nivolumab	PD1	Relapsed or refractory HL	2016
	Pembrolizumab	PD1	Refractory HL and PMBCL	2017
CAR T cells	Axicabtagene ciloleucel	CD19	DLBCL, transformed FL, PMBCL	2017
	Tisagenlecleucel	CD19	Relapsed or refractory LBCL	2018

DLBCL: diffuse large B-cell lymphoma; FL: follicular lymphoma; HL: Hodgkin lymphoma; LBCL: large B-cell lymphoma; NHL: non-Hodgkin lymphoma; PMBCL: primary mediastinal B-cell lymphoma

Myeloid-derived suppressor cells (MDSCs) have been shown to contribute to the immunosuppressive tumor microenvironment and tumor progression in B-cell lymphomas (Betsch et al., 2018). In particular, MDSCs may suppress T cell functions via expressing PD-L1, secreting IL-10/TGF-β, inducing T regulatory (Treg) development and expansion, and depleting certain amino acids (Serafini et al., 2008; Azzaoui et al., 2016; Roussel et al., 2017). Increased MDSCs are observed in various B-cell lymphomas, including HL, DLBCL and follicular lymphoma (FL), which correlates with poor prognosis and overall survival of patients (Roussel et al., 2017; Betsch et al., 2018). Hence, MDSCs may also play a role in causing resistance to cancer immunotherapy in B-cell lymphomas.

## HDAC and HDAC Inhibitors in B-Cell Lymphomas

Histone acetylation level is determined by a dynamic equilibrium between histone acetyl transferases (HATs) and histone deacetylases (HDACs); namely, HATs add acetyl groups to histone lysine residues while HDACs remove them. HDACs consist of a large family of proteins categorized into five groups, class I (HDAC 1, 2, 3, 8), class IIa (HDAC 4, 5, 7, 9), class IIb (HDAC 6, 10), class III (Sirtuins) and class IV (HDAC 11). Dysregulation of histone acetylation can lead to aberrant gene expression, which can activate oncogenes, inactivate tumor suppressors, inhibit programed cell death and mediate immune evasion, ultimately resulting in tumor progression (Kroesen et al., 2014). Aberrant HDAC expression occurs in both solid tumors and hematological cancers, including B-cell lymphomas (Ropero and Esteller, 2007; Zain and O’Connor, 2010; Lee et al., 2014). Prior findings show that dysregulation of histone acetylation contributes to lymphomagenesis, particularly in GC-derived lymphomas (Sermer et al., 2019). Constitutive expression of HDAC 9 throughout B cell development in mice causes splenic marginal zone lymphoma and lymphoproliferative disease that progresses towards aggressive DLBCL (Gil et al., 2016). Taken together, HDACs may serve as potential targets of chemical intervention for treating B-cell lymphomas (Table 2).

**Table 2:** The application of HDAC inhibitors to B-cell lymphoma (BCL)

Names	Potential mechanisms of action	Target (HDAC)	Subtype
Apicidin	Upregulate MHC I and II	HDAC3/6	DLBCL
Belinostat	Induce cell death, upregulate MHC I and II	HDAC1/2/3/4/5/6/7/8/9/10/11	DLBCL, MCL, FL, TL, HG-BCL, BL, PMBCL
Citarinostat	Induce cell death	HDAC6	FL, MCL, HL
CI-994	Induce cell death	HDAC1/3	PEL, canine BCL
Entinostat	Induce cell death, upregulate MHC I and II, CD20, reduce MDSC	HDAC1/2/3/10	HL, NHL, DLBCL, BL
MC1568	CD1d-mediated antigen presentation	HDAC4/5/6/7/9/10	MCL
Mocetinostat	Induce cell death, upregulate PD-L1	HDAC1/2/3/11	HL, DLBCL, FL
MPT0E028	Induce cell death	HDAC1/2/6	BL
OKI-5/OKI-179	Induce cell death, upregulate PD-L1, MHC I and II	HDAC1/2/3/6/8/10/11	DLBCL, murine BCL
Panobinostat	Induce cell death, CD1d-mediated antigen presentation, upregulate PD-L1, inhibit CD4/8 T cells	HDAC1/2/3/4/5/6/7/8/9/10/11	HL, DLBCL, MCL, BL, murine and canine BCL
RGFP966	Induce cell death, upregulate MHC I, CD80, CD86, PD-L1	HDAC3	DLBCL, murine BCL
Ricolinostat	Induce cell death	HDAC6	DLBCL, MCL, FL
Romidepsin	Induce cell death, upregulate MHC I, CD80, CD86, CD20, NKG2D ligands	HDAC1/2	BL, DLBCL, MCL, murine BCL
SBHA	Induce cell death	HDAC1/3	MCL, PEL, canine BCL
Scriptaid	Induce cell death	HDAC1/3/8	DLBCL, canine BCL
Sodium butyrate	Induce cell death, upregulate MHC I and II	HDAC1/2/3	BL, FL, PEL, DLBCL
Trichostatin A	Induce cell death, upregulate MHC I and II, MHC II-mediated antigen presentation, CD1d-mediated antigen presentation, upregulate PD-L1, activate Tregs	HDAC1/2/3/6/8/10/11	DLBCL, MCL, murine and canine BCL
Tubacin	Induce cell death, activate Tregs	HDAC6	BL, canine BCL
Tubastatin A	Induce cell death, activate Tregs	HDAC6	DLBCL, MCL
Valproic acid	Induce cell death, upregulate MHC I and II, CD80, CD86, PD-L1, CD20, activate Tregs	HDAC1/2	BL, DLBCL, MCL, murine BCL
Vorinostat	Induce cell death, upregulate PD-L1, activate Tregs	HDAC1/2/3/5/6/8/9/10/11	FL, DLBCL, HL, MCL, iNHL, BL, murine and canine BCL

BL: Burkitt lymphoma; DLBCL: diffuse large B-cell lymphoma; FL: follicular lymphoma; HG-BCL: high grade B-cell lymphoma; HL: Hodgkin lymphoma; iNHL: indolent NHL; MCL: mantle cell lymphoma; NHL: non-Hodgkin lymphoma; PEL: primary effusion lymphoma; PMBCL: primary mediastinal B-cell lymphoma; TL: transformed lymphoma

To date, four HDAC inhibitors, vorinostat, romidepsin, panobinostat, and belinostat, have been approved by the United States Food and Drug Administration and are used for hematologic cancers in clinic (Yoon and Eom, 2016). In addition, several new HDAC inhibitors have been tested in clinical trials against different types of tumors, however, single treatment regimens have poor therapeutic effect on solid tumors (Thurn et al., 2011). Despite the success of FDA-approved HDACi, each has intrinsic liabilities including poor isoform selectivity, marginal potencies towards relevant isoforms, narrow therapeutic indices and/or non-oral delivery routes. These drawbacks have spurred a continuous search for alternatives with improved biological, physiochemical and therapeutic properties. Luesch and colleagues reported a natural product, largazole, as a potent class I HDACi (Taori et al., 2008; Ying et al., 2008); however, largazole has relatively poor physiochemical properties and is not amenable to large-scale chemical manufacturing. Thus, a lead optimization program was initiated that resulted in the discovery of next generation largazole derivatives OKI-005 and OKI-179 (Liu et al., 2013). Our recent studies show that combined treatment of OKI-179/anti-PD1 significantly inhibited growth of B-cell lymphomas refractory to PD1-blockade; furthermore, sensitivity to single or combined treatment required tumor-derived MHC class I, and positively correlated to MHC class II level (Wang et al., 2019). Here, we focus on the rationale and therapeutic promise of combining HDAC inhibitors with immunotherapy in the treatment of B-cell lymphoma.

## Cytotoxicity of HDAC Inhibitors

HDAC inhibitors can suppress tumor growth via multiple mechanisms (Table 2). Mocetinostat, a class I/IV HDAC inhibitor, induces cell apoptosis and cell cycle arrest, and downregulates Bcl-2 in HL cell lines (Huang et al., 2018). A pan-HDAC inhibitor, panobinostat, can induce cell death, autophagy, and an increase of MICA/B, ligands of NK cell receptors in HL cell lines (Klein et al., 2013). Entinostat, a class I HDAC inhibitor, decreases Bcl-XL levels and induces caspase-dependent/independent apoptosis in B-cell lymphomas (Frys et al., 2015), while entinostat induces caspase-dependent apoptosis in B-cell chronic lymphocytic leukemia cells (Lucas et al., 2004). In addition, the anti-tumorigenic effects of HDAC inhibitors can be mediated by the activation of TRAIL and Fas signaling pathways in leukemia (Insinga et al., 2005). Seven HDAC inhibitors, including CI-994, panobinostat, SBHA, vorinostat, scriptaid, trichostatin A and tubacin, exhibit dose-dependent inhibitory effects on proliferation of a canine B-cell lymphoma line (Dias et al., 2018). Another pan-HDAC inhibitor, MPT0E028, shows a potent HDAC inhibitory effect, that leads to increased apoptosis, and prolongs the overall survival of recipient mice bearing human B-cell lymphoma in a xenograft model (Huang et al., 2015).

Our studies also demonstrate cytotoxic effects of HDAC inhibitors on B-cell lymphomas (Wang et al., 2019). Both OKI-005 and OKI-179 (class I, IIb, IV HDAC inhibitors) cause cell cycle arrest, apoptosis and growth inhibition in multiple murine and human B-cell lymphoma lines (Wang et al., 2019). Although OKI-179 displays direct cytotoxic effects on lymphomas, our studies highlight its immunological effects because we find that tumor-derived MHC class I is absolutely required for the therapeutic effects of OKI-179 single agent treatment (Wang et al., 2019). Thus, our data demonstrate that the therapeutic effects of OKI-179 mainly depend on its ability to modulate immune responses, instead of its anti-proliferative or pro-apoptotic role (Wang et al., 2019).

## Immune Modulation of B-Cell Lymphomas by HDAC Inhibitors

Since MHC class I expression is often reduced by epigenetic mechanisms in cancers, HDAC inhibitors can upregulate MHC class I in various types of cancers (Grunewald et al., 2018). In DLBCL cell lines, HDAC inhibitors (trichostatin A, sodium butyrate, apicidin and entinostat) induce MHC class I expression (Cycon et al., 2013). Valproic acid (class I HDAC inhibitor), romidepsin (HDAC1/2 inhibitor) or RGFP966 (HDAC3 inhibitor) upregulates MHC class I expression and costimulatory molecules, such as CD80 and CD86, in B-cell lymphomas (Deng et al., 2019). Histone deacetylation typically induces a closed chromatin state at the MHC class II promoters, resulting in downregulation of MHC class II in tumors. HDAC inhibitors, including trichostatin A, sodium butyrate, apicidin, valproic acid and entinostat, increase MHC class II expression in DLBCL (Cycon et al., 2013). In mantle cell lymphoma (MCL), trichostatin A treatment enhances MHC class II-mediated antigen presentation, even though MHC class II expression remains unaltered (Tiper and Webb, 2016). *Crebbp* deficiency was shown to promote B-cell lymphomagenesis with a HDAC3-mediated enhancer repression signature, including the downregulation of MHC class II (Jiang et al., 2017). Inhibiting HDAC3 restored the expression of MHC class II in *Crebbp*-deficient cells, thus enhancing T cell proliferation (Jiang et al., 2017). Our studies also show OKI significantly enhance MHC class I and class II expression in G1XP lymphoma both *in vitro* and *in vivo* (Wang et al., 2019). However, OKI-179 upregulated HLA-DP, DQ and DR in OCI-Ly3 cells, but not in OCI-Ly1, OCI-Ly7 and SU-DHL-16 cells (Wang et al., 2019). Thus, different human B-cell lymphoma lines respond differentially to OKI-179 treatment in terms of MHC upregulation (Wang et al., 2019).

HDAC inhibitors affect not only classical MHC expression but also non-classical MHC expression such as CD1d. CD1d mediates antigen presentation to NKT cells, a subset of T cells that recognize lipid antigens. In MCL, treatments with trichostatin A, panobinostat or MC1568 rapidly enhances CD1d-mediated antigen presentation (Tiper and Webb, 2016). Mechanistically, HDAC2 can bind to the promoter of CD1d and HDAC2 knockdown in tumor cells results in a significant increase of CD1d surface expression, thus enhancing CD1d-mediated immune recognition and activation of NKT cells (Tiper and Webb, 2016). Trichostatin A can also enhance CD1d antigen presentation via abrogating the secretion of IL-10 by tumor cells (Tiper and Webb, 2016). α-GalCer is a glycolipid antigen presented by CD1d and a potent activator of iNKT cells. In a preclinical B-cell lymphoma murine model, the combination of α-GalCer and vorinostat significantly reduced tumor burden and increased recipient survival compared to single agent treatment (West et al., 2013).

PD-L1 expression in cancer cells has been associated with intrinsic aggressive features, which provides a rationale for developing new drugs to target PD-L1 expression directly or to potentiate therapeutic effects by combining drugs with PD-1/PD-L1 inhibitors. In this regard, multiple HDAC inhibitors can modulate PD-L1 expression. Class I HDAC inhibitors (entinostat, mocetinostat, panobinostat and belinostat) have been shown to generate robust and durable upregulation of PD-L1 and PD-L2 in tumor cells; however, this upregulation was not observed upon inhibiting Class IIa HDACs or HDAC 6 with rocilinostat, nexturastat A or PCI34051 (Woods et al., 2015). Mocetinostat increases PD-L1 expression in multiple HL cell lines (Huang et al., 2018). Pan-HDAC inhibitors, such as vorinostat, TSA and panobinostat, induce PD-L1 expression in B-cell lymphomas (Deng et al., 2019). Selective HDAC3 inhibitors (e.g., RGFP966) also upregulated PD-L1 expression because HDAC3 was shown to repress PD-L1 transcription in B-cell lymphomas (Deng et al., 2019). We showed that OKI-179 also upregulated PD-L1 expression in B-cell lymphomas but not in primary B cells (Wang et al., 2019). Notably, OKI-179 exhibits potent inhibitory activities on HDAC 1, 2 and 3 (Wang et al., 2019), consistent with the notion that HDAC3 is a crucial repressor of PD-L1 transcription (Deng et al., 2019).

Prior studies show that tumor-derived PD-L1 is not required for the efficacy of anti-PD-L1 treatment because host myeloid cells still express PD-L1 that is essential for the response to anti-PD-L1 (Tang et al., 2018). It remains to be determined whether altering tumor-derived PD-L1 will affect the efficacy of combined treatment of OKI-179/anti-PD1 in our B-cell lymphoma model (Wang et al., 2019). Based on these prior studies, we suggest that HDACi-mediated PD-L1 upregulation may explain why these agents generally fail to treat cancers as a single agent. Our studies may provide novel insights into why HDACi alone failed and why there should be a renewed emphasis on the combined therapies of HDACi and ICIs.

Downregulation of CD20 is a clinical issue that leads to decreased efficacy of anti-CD20-based therapeutic regimens. Entinostat increases the expression of CD20 and adhesion molecules in multiple B-cell lymphomas, thereby sensitizing B-cell lymphomas to the treatment of anti-CD20 monoclonal antibodies (Frys et al., 2015). Furthermore, inhibiting HDAC 6 significantly upregulates CD20 expression in B cell tumors and enhances the efficacy of anti-CD20 monoclonal antibodies (Bobrowicz et al., 2017). HDAC inhibitors, valproic acid and romidepsin, can transactivate the *CD20* gene via promoter hyperacetylation and Sp1 recruitment (Shimizu et al., 2010); therefore, both HDAC inhibitors increase CD20 expression in B-cell lymphoma lines and reduce the growth of B-cell lymphomas synergistically with anti-CD20 monoclonal antibodies. Phase II trials were performed to combine HDAC inhibitors (panobinostat or vorinostat) and rituximab (an anti-CD20 antibody) in patients with DLBCL or indolent NHL (Chen et al., 2015; Assouline et al., 2016). CD20 CAR T cells are being tested in clinical trial for NHL; however, tumor cells can lose or downregulate CD20 expression (Shah et al., 2019). We suggest that combining HDAC inhibitors with CAR T cells may provide another novel approach to overcome resistance to CAR T cell therapy.

Apart from alterations in antigen expression, cancer cells may acquire resistance to CAR T cell therapy by dysregulating their apoptotic machinery, such as apoptotic pathway mediated by tumor necrosis factor-related apoptosis-inducing ligand (TRAIL) (Torres-Collad and Jazirehi, 2018). When human NHL cell lines became resistant to CD19 CAR T cells, adding HDAC inhibitors, vorinostat (a.k.a. SAHA) or panobinostat, largely reversed their resistance to CD19 CAR T cells (Torres-Collad and Jazirehi, 2018). Studies also indicate that vorinostat sensitized CD19 CAR T cell-resistant Ramos cells to TRAIL-mediated killing (Torres-Collad and Jazirehi, 2018), although the detailed mechanisms remain to be defined. HDAC inhibitors including vorinostat have been shown to upregulate the expression of death receptor 5 (DR5), a receptor for TRAIL, on human cancer cells (Nakata et al., 2004). Thus, we suggest that HDAC inhibitors may function by modulating death receptor expression (e.g., DR5) in cancer cells and enhance their sensitivity to players in extrinsic apoptotic pathways (e.g., TRAIL). In addition, HDAC inhibitors have been reported to improve the function of CAR NK cells (Chu et al., 2015; Chu et al., 2017). Romidepsin significantly enhances the expression of NKG2D ligands in cancer cells that can activate NKG2D expressed in NK cells, thereby enhancing the cytotoxicity of anti-CD20 CAR NK cells to romidepsin-treated Burkitt lymphoma cells (Chu et al., 2015; Chu et al., 2017). In humanized Raji xenograft NSG mice, combined treatment with romidepsin and anti-CD20 CAR NK cells achieves a better therapeutic efficacy than single agent treatment (Chu et al., 2015; Chu et al., 2017).

## Effects of HDAC Inhibitors on Immune Cells

HDAC inhibitors not only affect cancer cells but also modulate immune cell functions. Some HDAC inhibitors have direct effects on T cells. Trichostatin A causes a rapid decline in cytokine expression, arrests cell cycle at G1 phase and induces apoptosis of CD4 T cells (Moreira et al., 2003). However, another study shows that trichostatin A can inhibit apoptosis of tumor infiltrating CD4 T cells by suppressing NFAT1-regulated Fas ligand expression on activated CD4 T cells and thereby enhance anti-tumor immune responses (Cao et al., 2015). Tregs are responsible for inhibiting or regulating immune responses by suppressing effector T cells. Previous studies show that exposure to multiple HDAC inhibitors enhances the suppressive function of Tregs, correlative to increased FOXP3 and CTLA-4 expressions in Tregs (Tao et al., 2007; Akimova et al., 2010). Further data show that inhibiting HDAC6, HDAC9 or the class III HDAC Sirtuin-1 promotes the suppressive activity of Tregs (Tao et al., 2007; Beier et al., 2011; de Zoeten et al., 2011). The knockout of HDAC5 reduces the differentiation and suppressive function of Tregs (Xiao et al., 2016). However, CD8 T cells without HDAC5 have a reduced ability to produce IFN-γ (Xiao et al., 2016). Our study shows that OKI, along with vorinostat, has no effect on the proliferation and activation of CD4 and CD8 T cells (Wang et al., 2019). In contrast, panobinostat significantly inhibits the proliferation and activation of CD4 and CD8 T cells at a low concentration (Wang et al., 2019), suggesting that such low therapeutic windows probably make it unsuitable for combinations with ICIs. Due to the cytotoxicity of HDAC inhibitors and their variable effects on Tregs, it is critical to consider their direct effects on T cells in the application of HDAC inhibitors for cancer immunotherapy.

MDSCs are a myeloid population, capable of suppressing various T-cell functions in the tumor microenvironment. In HDAC11-defcient mice, MDSCs are highly suppressive and transplanted T cell lymphomas are more aggressive compared to wild-type controls (Sahakian et al., 2015). Adding entinostat to PD-1 and CTLA-4 blockade significantly reduces the number of MDSCs, leading to enhanced tumor control and reduced metastasis (Kim et al., 2014); however, it remains unknown which HDACs mediate such effects. Based on its biochemical profile, entinostat inhibits the activity of HDAC 1, 2, and 3 but not HDAC 11 (Wang et al., 2019). Hence, it may be also important to consider the effects of HDAC inhibitors on MDSCs when using them for cancer immunotherapy.

## Perspectives and Future Directions

Lack of selectivity of many HDAC inhibitors may contribute to toxicities against healthy cells in clinical applications and trials. Nonselective HDAC inhibitors target multiple HDACs, which usually cause serious adverse events, including thrombocytopenia, fatigue and diarrhea (Gryder et al., 2012). In addition, not all HDACi are created equal and their net effects are highly dependent on the specific inhibitors used and the HDACs they target (Kroesen et al., 2014; McCaw et al., 2017). Class I HDAC inhibitors have been shown to enhance the function of NK and CD8 T cells, reduce the number and function of Tregs, and in turn, result in anti-tumor immune responses in multiple tumor models (Kroesen et al., 2014). However, class II HDAC inhibitors directly enhance the immunosuppressive function of Tregs (Kroesen et al., 2014). Furthermore, inhibiting HDAC2 (class I HDAC) enables highly immunosuppressive tumor-induced MDSCs differentiate into macrophages and dendritic cells, which stimulates the immune system to target tumor cells (Youn et al., 2013). In contrast, pan-HDAC inhibition results in the accumulation of a pool of undifferentiated myeloid cells, which limits anti-tumor immune responses (Rosborough et al., 2012). Moreover, a pan-HDAC inhibitor, panobinostat, is very toxic to T cells, although it can also induce MHC expression (Wang et al., 2019). Since an intact immune system is required for the anti-B-cell lymphoma activities of HDAC inhibitors (West et al., 2013), it is important to consider isoform selectivity for the rational design of combinatorial immunotherapy using HDAC inhibitors; for instance, class I HDAC inhibitors may be more appropriate to be combined with ICIs than class II or pan-HDAC inhibitors. Taken together, it is thereby ideal to seek HDAC isoform-specific inhibitors, which exhibit optimal biochemical features that allow their targeted use to enhance apoptosis of tumor cells, increase tumor immunogenicity, and reverse immunosuppressive tumor environments, thereby favoring anti-tumor immune responses.
